# Small Angle X-ray and Neutron Scattering: Powerful Tools for Studying the Structure of Drug-Loaded Liposomes

**DOI:** 10.3390/pharmaceutics8020010

**Published:** 2016-03-28

**Authors:** Emanuela Di Cola, Isabelle Grillo, Sandra Ristori

**Affiliations:** 1 Laboratoire Interdisciplinaire de Physique (LIPhy), Université Grenoble-Alpes, CNRS-UMR 5588, 140 rue de la Physique, 38402 Saint-Martin-d’Hères, France; phyedc@gmail.com; 2 Institut Laue-Langevin (ILL) DS/LSS, CS 20156-38042 Grenoble Cedex 9, France; grillo@ill.fr; 3 Dipartimento di Scienze della Terra, Università degli Studi di Firenze, Via della Lastruccia 3, 50019 Sesto Fiorentino, Italy

**Keywords:** small angle scattering, liposome structure, SAXS, SANS, drug loading, ASAXS

## Abstract

Nanovectors, such as liposomes, micelles and lipid nanoparticles, are recognized as efficient platforms for delivering therapeutic agents, especially those with low solubility in water. Besides being safe and non-toxic, drug carriers with improved performance should meet the requirements of (i) appropriate size and shape and (ii) cargo upload/release with unmodified properties. Structural issues are of primary importance to control the mechanism of action of loaded vectors. Overall properties, such as mean diameter and surface charge, can be obtained using bench instruments (Dynamic Light Scattering and Zeta potential). However, techniques with higher space and time resolution are needed for in-depth structural characterization. Small-angle X-ray (SAXS) and neutron (SANS) scattering techniques provide information at the nanoscale and have therefore been largely used to investigate nanovectors loaded with drugs or other biologically relevant molecules. Here we revise recent applications of these complementary scattering techniques in the field of drug delivery in pharmaceutics and medicine with a focus to liposomal carriers. In particular, we highlight those aspects that can be more commonly accessed by the interested users.

## 1. Introduction

**Figure 1:**
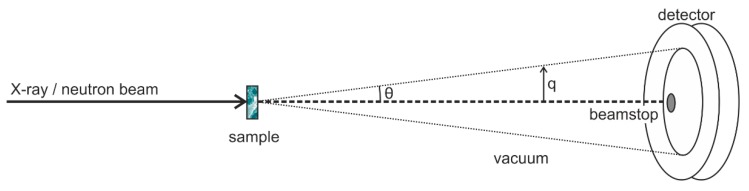
Layout of a conventional small angle X-ray or neutron scattering setup. Adapted from [6].

**Figure 2:**
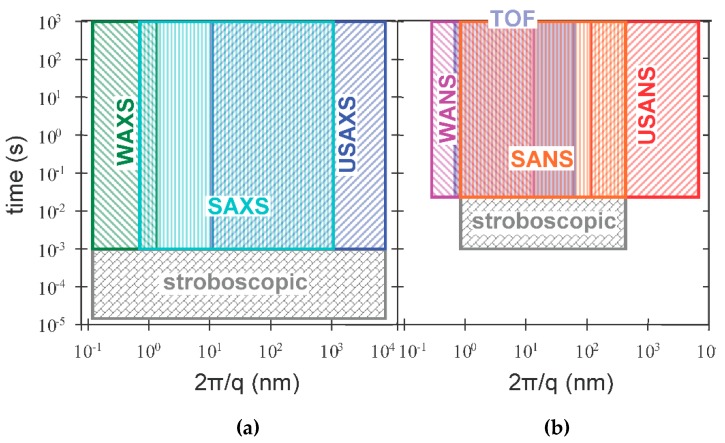
Time and length scale provided by small-angle X-ray and neutron scattering experiments (panel (**a**) and (**b**), respectively).

Small-angle X-ray and neutron scattering (SAXS and SANS, respectively) measurements are conceptually very simple and the basic principles are similar to those of light scattering, which is now routinely used in biological and pharmaceutical labs to estimate the size distribution of formulations with particles in the submicron domain [1]. Though there are common underlying principles and methods for extracting structural information from X-ray or neutron scattering data, there are fundamental differences in the way X-rays and neutrons are produced and how they interact with the scattering medium [2,3,4]. In the case of X-rays, the scattering originates from electrons, and it is nearly independent of the incident wavelength, except in the close vicinity of the absorption edge of the constituent elements. In the latter case, the technique offers an opportunity for contrast variation measurements [2,5], as will be illustrated in Section 2.2. Figure 1 shows the scattering geometry of a conventional SAXS and SANS experimental setup [6]. In the case of a synchrotron X-ray source, a highly collimated and monochromatic beam impinges on a sample and the scattered intensity in the forward direction is collected by a two-dimensional detector. The transmitted primary beam is fully absorbed by a beamstop placed in front of the detector, and the flight path before and after the sample is commonly in vacuum in order to avoid absorption and scattering by air. In modern instruments, the beamstop is often equipped with intensity monitors to record the transmission simultaneously with the scattering pattern. Subsequently, the two-dimensional scattering pattern is radially integrated to provide the one-dimensional scattering function *I*(*q*), where *q* is the length of the scattering vector (*i.e.*, the momentum transfer), defined by *q*$=\frac{4\pi }{\lambda }\sin\frac{\theta }{2}$, λ being the incident wavelength (typically of the order ~1 Å) and θ the scattering angle. The scattering vector *q* provides the length scales probed by the experiment. X-ray scattering can give information over a wide range of scattering angles from ultralow angles (USAXS), small angles (SAXS), and wide angle (WAXS) [2,3]. In modern synchrotron SAXS instruments [7] the *q* range can ultimately cover more than four orders of magnitude, 0.001 nm^−1^ < *q* < 50 nm^−1^, corresponding to real space dimensions from the µm down to the nm range (Figure 2a). Reviews of experimental aspects of the technique and descriptions of the different types of instruments for X-ray scattering can be found elsewhere [3,6,8,9].

Unlike X-rays, neutrons interact with the nucleus of the atom. The scattering length of atoms varies in a random way and can even be negative. The main feature is the difference in scattering length between hydrogen (−3.74 × 10^−15^ m) and its isotope deuterium (6.67 × 10^−15^ m). The contrast variation technique, either by a specific deuteration of molecules or the use of H_2_O/D_2_O solvent mixture, is a powerful method for the study of complex systems made of several shells or components, such as, for instance, pure or loaded liposomes. Moreover, neutrons are non-destructive and radiation damage to the samples is not an issue as it could be in the case of X-rays from synchrotron sources.

The two main sources of neutrons are steady-state reactors and spallation sources. In the first case, neutrons are continuously produced by fission processes. In the second case, a pulsed neutron beam (typically with 25 or 50 Hz frequency) is generated by the collision of high-energy protons, which chop off heavy atoms.

For most steady-state instruments, the wavelength is selected through a mechanical velocity selector, which consists of a rotating drum with helically curved absorbing slits at its surface. The wavelength can be varied between about 4.6 and 40 Å for selecting the rotation speed. Instruments at pulsed spallation sources use the time-of-flight (TOF) method. A system of choppers (between two and four) are used to create neutron pulses. The wavelength is calculated from the time it takes for a neutron pulse to arrive on the detector. The main advantage of a TOF instrument is to cover a massive dynamic *q*-range, *q*_max_/*q*_min_, up to 100, in only one instrument setting (Figure 2b). Some instruments such as D33 at the Institute Laue Langevin (ILL, Grenoble, France), have the possibility to work with these two modes [10].

SAXS and SANS are therefore complementary techniques that, when combined, allow a fine structural description of the particles under investigation.

**Figure 3:**
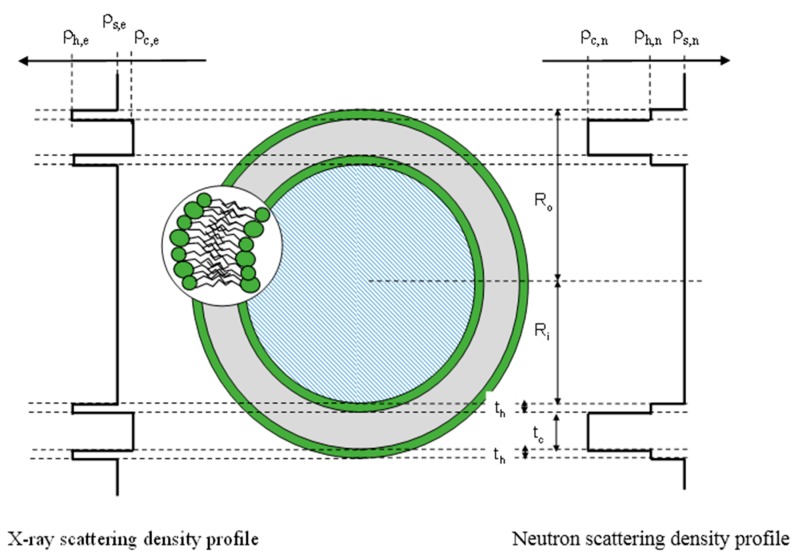
Comparison of the scattering length density profile of liposome for X-ray (**left**) and neutron (**right**). Adapted from [11].

**Figure 4:**
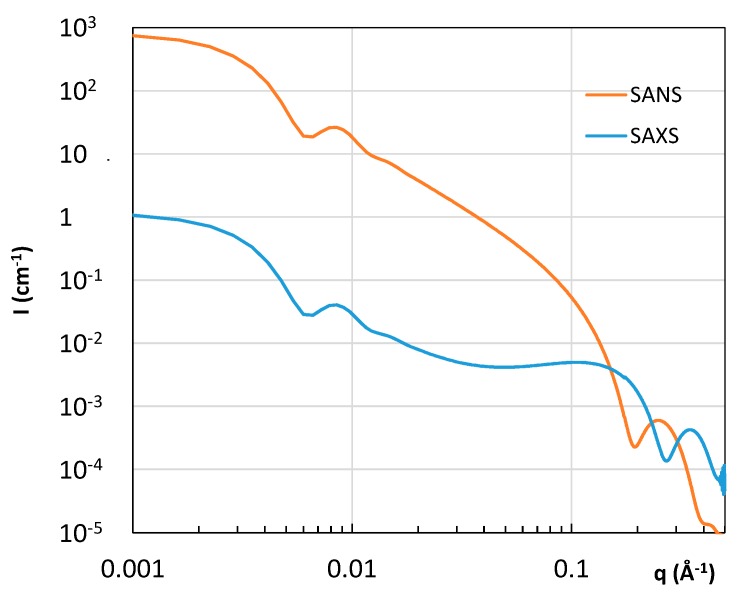
Calculated small-angle X-ray scattering (SAXS) and small-angle neutron scattering (SANS) intensity for a suspension of 1% of liposomes. The SLD profile is shown in Figure 3. *R*_i_ = 450 Å, *t*_h_ = 5.4 Å; *t*_c_ = 28 Å; ρ_h_,_n_ = 3.5 × 10^10^ cm^−2^; ρ_c_,_n_ = −0.208 × 10^10^ cm^−2^; ρ_s_,_n_ = 6.4 × 10^10^ cm^−2^; ρ_h_,_e_ = 13.2 × 10^10^ cm^−2^; ρ_c_,_e_ = 7.6 × 10^10^ cm^−2^; ρ_s_,_e_ = 9.33 × 10^10^ cm^−2^.

In a simple picture, a single bilayer liposome can be described with a model of a core surrounded by three concentric shells, and the scattering length profile both for X-ray and neutron is reported in Figure 3. The scattered intensity obtained by SAXS and SANS is compared in Figure 4. In the case of X-rays, the scattering length density of the hydrophilic part (ρ_h,e_) is higher than the scattering length density of the aqueous solvent (ρ_s,e_), this latter being only slightly above the scattering length density of the hydrophobic core (ρ_t,e_). With neutrons, when liposomes are prepared in a deuterated buffer, a good contrast is obtained for the hydrophobic tail, whereas the head group is almost not visible. Consequently, X-rays are more sensitive to the polar layer, whereas neutrons provide information on the hydrophobic layer. Drug molecules, according to their water affinity, will preferentially be inserted into one liposome compartment, modifying its average scattering length density and thus the scattered intensity.

For both techniques the analysis of the scattering patterns is strongly linked to the data quality. Therefore, sufficient statistics at large angles must be ensured to accurately subtract the scattering signal coming from the solvent. This is mandatory to evidence subtle changes that might occur in the bilayer. It should be pointed out here that in SANS, the attempt to separate the incoherent signal for the relevant coherent signal using polarization and analysis are still scarce, and the results are not optimized [12,13]. The decrease of the incident flux by a factor of 10 under polarized conditions, and the need to repeat the experiment at different polarization states and contrast requires a very long acquisition time (typically a few hours). Moreover, the ^3^He box analysis places after the sample reduced the solid angle and the maximum *q* at about 0.1 Å^−1^, below the relevant *q* range for the bilayer characterization. Finally, a good angular resolution of the beam is needed to avoid smearing out the characteristic oscillations coming from the intrinsic structural parameters such as the radius and the bilayer thickness. Finally, absolute scale units are the guaranty for a quantitative description of the system.

In this paper we mainly focus on the recent applications of SAXS and SANS for the characterization of lipidic self-assembled soft nano-structures. Experiments performed both under equilibrium and non-equilibrium conditions will be illustrated in the following examples. The cited studies are only a representative selection of the current structural investigations by means of SAXS and SANS in the field of drug delivery. The scope is meant to give to the reader a flavor of the broad range of information that can be provided by the combinations of these two techniques. For the sake of completeness, lipid membranes can be also studied on solid supports or at the water/air interface by reflectometry and grazing incidence (GID) X-ray or neutron scattering. Like their solution counterpart, these methods provide information on drug loading and diffusion across lipid layers [14,15,16]. However, due to limited space, this subject will not be discussed herein and the interested reader is referred to specialized texts [17,18].

## 2. SAXS Applied to the Characterization of Liposomes

### 2.1. Studies under Equilibrium Conditions

The main goal of drug delivery is the development of carrier systems (polymeric micelles, liposomes, dendrimers, magnetic nanoparticles) capable of releasing drugs in a controlled manner. Therefore, most of the experiments are designed to correlate the pharmacological efficiency to their inner structure and to the role played by the physical and chemical parameters of the complexes, *i.e.*, investigate the function-structure relationship of the materials. In recent years, SAXS has been shown to provide valuable information to study the structure in self-assembled soft matter systems suitable for drug delivery [19,20,21]. SAXS makes it possible to reconstruct the structure of drug carriers alone or loaded with drugs in a natural aqueous environment [22]. The dimensions, shape and electron density maps can be thus obtained for biodegradable carriers [23] (Figure 3 and Figure 4). SAXS investigations can also help to localize the active guest molecules in the self-assembled nanostructures and therefore study the effect of drug loading on the inner morphology (see Section 2.2). In addition, SAXS can provide information about the structural arrangement of the loaded drugs, which is strictly related to the pharmacological activity. Recently, Schilt *et al.* have reported a throughout investigation, based on SAXS and WAXS, on a series of PEGylated liposomes loaded with doxorubicin, a system that has already been approved for clinical applications [14]. The accurate modelling of the scattering curves made it possible to establish that the drug inside liposomes adopts a crystalline or amorphous state according to chemical derivatization (*i.e.*, sulphate *versus* methanesulphonate, respectively). The authors also found that the structure of doxorubicin is essentially the same in the Doxil^®^ formulation and in its generics.

PEGylated liposomes (also called sterically stabilized liposomes) are a class of widely used nanovectors for *in vivo* treatment, since the polymer layer on their surface is able to create a barrier for opsonization, thus allowing for longer circulation time. However, there might be some limiting factors to their application related to their large size or to decreased efficacy if the drug action is exerted through surface loading and specific cell recognition. In recent years, synchrotron SAXS has given a relevant contribution to the fine structure investigation of PEGylated liposomes [24,25,26,27].

Among the routes exploited for drug delivery, complexes of nucleic acids and cationic lipids (lipoplexes) have received great attention in the last years as non-toxic gene carriers that can effectively deliver molecules into specific cell types, including cancerous cells [28]. Since the pioneering work of Safinya and coworkers [29,30], SAXS techniques have been extensively used over the years to solve the nanostructure of lipoplexes under equilibrium conditions and to relate the structural features to their transfection efficiency [31,32,33,34]. Nowadays, efforts are devoted to investigate the structural modifications that lipoplexes can undergo upon *in vivo* administration, that consequently affect the delivery efficiency. In particular, SAXS and SANS have been largely employed to study the role played by biological fluids and cellular lipids in the biological membranes which might act as barrier for transfection [35,36,37,38,39].

Beside lipoplexes technology, alternative routes for the design of target and control molecules are offered currently by supramolecular organic frameworks (SOF), which can generate channels that allow guest absorption [40], magneto-responsive microgels [41] or solid lipid nanoparticles [42]. For instance, the fruitful combination of SAXS and WAXS studies on solution-phase self-assembly of a 3D periodic porous SOF in water, using the encapsulation of cucurbit[8]uril (CB[8]) for two 4-(4-methoxyphenyl)pyridin-1-ium (PP) units, has been recently reported [40]. It has been shown that the periodic SOF in solution acts as 3D-ordered cationic supramolecular polyelectrolyte that can attract a variety of anionic organic molecules, including dyes, drugs, peptides, nucleic acids and poly(amidoamine) (PAMAM) dendrimers, in both solution and solid states. The attracted drugs can be selectively released into water in an acidic medium.

### 2.2. Localisation of Guest Molecules in Drug Loaded Liposomes

The localization of guest molecules (targeting agents, drugs) inside nanocarrier systems (liposomes, vesicles, nanoparticles, *etc.*) is of primary importance for the optimization of their composition. Moreover, liposomes are often studied as model systems mimicking biological membranes because of their characteristic double lipid layer. If a guest molecule, such as a drug agent, is added to the liposomes, their characteristic structure can be perturbed depending on the concentration of the added component. Therefore, it is important to localize the drug molecules in order to investigate their effect on biological membranes as well.

At this regard, anomalous small-angle X-ray scattering (ASAXS) has been shown to be a suitable tool to determine the location of guest molecules in liposomes. ASAXS represents a unique scattering technique which allows for the structural characterization of charged soft matter and biological relevant systems, using a contrast variation method when the atomic absorption edge of one of the constituent elements is at an accessible energy range. Whereas in a SANS experiment samples with same structural features but where different contents of deuterium are prepared, in an ASAXS experiment only one sample is needed and measurements are carried out around the X-ray absorption edge of the element of interest [43]. The X-ray scattering factors of the element are energy dependent and change significantly in the proximity of the absorption edge, providing the effect of contrast variation. Hence, the two methods are highly complementary and are often currently both required for a complete structural analysis of soft matter systems in order to reduce the ambiguity in models from a single scattering data profile. A detailed review of recent applications of ASAXS to charged soft matter systems is presented in [44]. Here, we would like to focus only on the application of the technique for the microstructure characterization in soft materials, reporting on recent studies on drug loaded liposomes. For instance, Varga *et al.* [45] employed ASAXS to localize 2,4-dibromophenol (DBP) in a multilamellar vesicle system constituted from 1,2-dipalmitoyl-*sn*-glycero-3-phosphatidylcholine (DPPC) and water. In the case of the higher DBP concentration, the distribution of the guest molecules was characterized by a sharp function, highlighting the role played by these molecules in the organization of the interdigitated phase. A similar experimental approach has been reported by Bóta *et al.* [46] in a comparative study of the location of copper ions and the dihalogenated phenol molecules at their low concentration in the DPPC–water liposomes. ASAXS could provide information about the structural features of the domains rich in ions or in organic guest molecules. It has also been shown that the guest molecules can be enriched in domains when present in low concentration. The domains rich in copper ions are dispersed randomly, while the halogenated phenols are inhomogeneously located in the chemically favorable lipid regions, inducing consequently phase separation. More recently, ASAXS was also employed to localize an epidermal growth factor receptor (EGFR) tyrosine kinase inhibitor drug inside a sterically stabilized PEGylated liposome (POPC) [47]. These studies illustrate the uniqueness of ASAXS to determine the spatial distribution of charge species with high precision and underline the versatility of the method for the structural characterization of a wide range of systems such as surfactant micelles or bilayer vesicles.

### 2.3. Studies under Non-Equilibrium Conditions: Time Resolved SAXS (Tr-SAXS)

The application of self-assembled lipid nanostructures as drug nanocarriers requires biocompatible systems which have a highly efficient cellular uptake and targeting [48,49]. Beside the investigation of the physical-chemical properties of these formulations, it is fundamental to reach a full understanding of the kinetic pathways of self-assembly to exploit such systems for controlled drug release, solubilization, emulsification, *etc.* However, the self-assembly of lipid bilayer structures [50,51], nano-particle-based drug delivery systems [52], and vesicle formation [50,53] are phenomena which typically occur on the sub-millisecond timescale. Hence, their investigation demands *in-situ* time-resolved experiments to follow the dynamics of the structural changes and the formation of possible intermediate states in a time window spanning the millisecond to the second range. Synchrotron X-ray radiation has significantly higher flux (typically between 10^12^ and 10^14^ photons/s) compared to the bench-top instruments, enabling scattering patterns to be obtained in the milliseconds range. Therefore, the high brilliance of an undulator source is exploited to probe simultaneously the microstructure and non-equilibrium dynamics of soft matter and related systems, from a few Angstroms to the micron scale, and down to the millisecond time range (Figure 2b) [54].

In this context, the rapid mixing technique (stopped-flow) in combination with Tr-SAXS have been extensively used for kinetic studies [50,54]. This experimental setup makes it possible to trigger the rapid mixing of two or more solutions at different ratio and flow rates, with a short initial deadtime (about 2 ms) in order to follow the early stage of a kinetic process. Time-resolved experiments can be performed in two ways: a real-time experiment wherein the scattering from the sample is followed continuously as a function of time (millisecond time resolution determined by read-out delay of detectors), or a stroboscopic experiment in which measurements are systematically repeated, varying the delay from mixing to collection of the first frame. This makes it possible to decrease the effective dead time between frames down to sub-milliseconds. Such collection methods rely on the precise synchronization and temporal reproducibility of the process [3,6,50]. However, the advent of the next generation of large-area pixel detectors will allow for the probing of equilibrium conditions in soft matter systems in the sub-millisecond range in real time.

Alternatively, the combination of microfluidics and SAXS provides also a powerful tool to investigate dynamic processes on a molecular level with a sub-millisecond time resolution. By controlling flow rates and channel volumes, time resolution can be translated to spatial resolution, which is easily exploited using focused X-ray beams. Due to a constant flow of material within the device, radiation can be controlled and acquisition times can be increased, allowing for better signal/noise ratios while maintaining temporal resolution. Recent improvements to SAXS, coupled with microfluidics devices applied to the study of biological specimens, can be found in [55].

The broad application of stopped-flow combined with SAXS and SANS has been recently reviewed [54]. Here, we limit to focus on some recent applications of the fruitful combination of Tr-SAXS with stopped-flow for the study of cationic nano-assemblies, such as liposomes, polymeric micelles and other nanoparticles of potential interest for pharmacological and biomedical exploitation.

**Figure 5:**
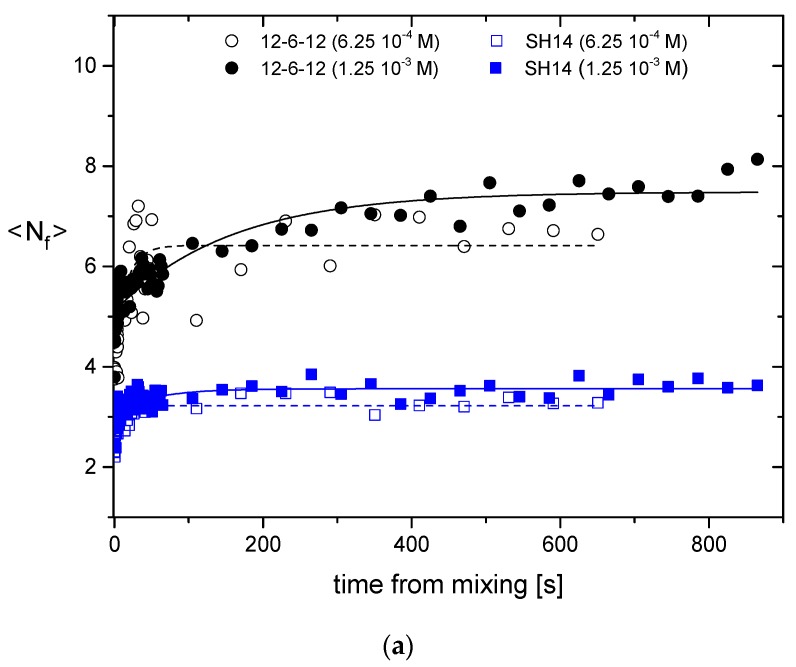

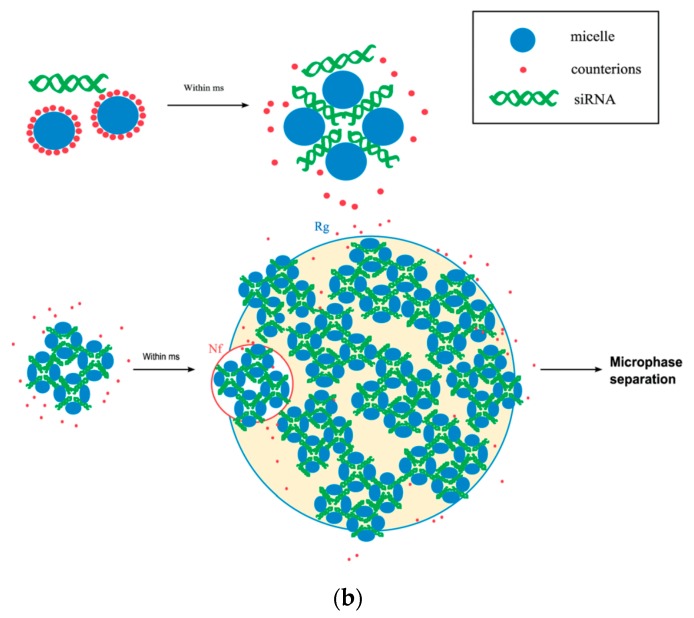
(**a**) Time evolution of the number of correlated layers in small interfering RNA (siRNA)/micelle complexes for surfactants 12-6-12 and SH14 at two different concentrations, for charge ratio CR = 1.25. (**b**) Schematic of the pathway of complex formation. (Reproduced from [56]. Copyright RSC 2014).

For instance, Falsini *et al.* [56] used Tr-SAXS to investigate the complexation of small interfering RNA (siRNA) with non-viral synthetic carriers (cationic micelles) for gene delivery. When combined with siRNA, non-viral vectors form complexes, whose structural features are in the nano- to microscale range. The state of the complex used for transfection strongly depends on the kinetic pathway followed during formation. Therefore, time-resolved SAXS experiments were employed to shed light on the mechanisms that determine the resulting structure and size. Micelles were obtained from two types of divalent cationic surfactants, *i.e.*, Gemini bis(quaternary ammonium) bromide with variable spacer length and a weak electrolyte surfactant (SH14) with triazine head. The time-resolved data indicate that complexation occurred within the first 50 ms, and the process of complex reorganization followed a first-order kinetics (see Figure 5a). In the new structural arrangement, siRNA and micelles were alternately layered in “sandwiches” with a repeat distance of 3–4 nm (Figure 5b). Aggregates containing SH14 were found less ordered compared to Gemini surfactants.

A similar experimental method was applied by [52] to follow the structural changes occurring during the uptake of DNA by cationic lipid nanocarriers (lipoplexes). Nanoparticles (NPs) were made of fusogenic lipid carriers with nano-channelled organization (cubosome nanoparticles) favorable for DNA upload. The rapid kinetics of complexation and assembly of these cubosome particles with neurotrophic plasmid DNA (pDNA) was revealed using a 4 ms time resolution. It was shown that pDNA upload into the highly hydrated channels of the cubosome carriers leads to a fast nanoparticle–nanoparticle structural transition and to lipoplex formation with tightly packed pDNA.

Tr-SAXS has been recently employed by [51] to study the structural changes during osmotic shrinkage of a pharmacologically relevant liposome drug delivery system. Sterically stabilized liposomes (SSLs) composed of hydrogenated soy phosphocholine (HSPC), cholesterol and distearoyl-phosphoethanolamine-PEG 2000 (DSPE-PEG 2000) prepared in a salt-free buffer were mixed with a buffer solution of 0.3M NaCl in 1:1 volume ratio using a stopped flow apparatus. The data provided a first description of the water permeability of liposomes of about 100 nm in size without the need of any additive tracer molecule. In addition, the applied method allowed for the parallel characterization of the bilayer structure, giving new insights into the process of osmotic shrinkage of SSLs. The changes in the liposome size and the bilayer structure were followed with a time resolution of 20 ms. The shrinkage of the SSLs was found to be a two-stage process, *i.e.*, an initial decrease of liposome size, followed by the deformation of spherical liposomes into lens-shaped ones.

For completeness, self-assembly in lipid systems can be also initiated by other routes, such as an abrupt variation of physical parameters like temperature or pressure [57]. Therefore, the associated structural changes can be studied using Tr-SAXS combined with dedicated pressure (P-jump) [58] or temperature (T-jump) [59,60] sample environments. Lipid systems are pressure and temperature sensitive and, in general, they easily undergo phase transformations under changes in environmental conditions [42]. Tr-SAXS combined with a rapid pressure jump across the phase boundary has proved to be extremely useful in identifying intermediate structures with a relative short lifetime in lipid phase transitions [58]. An overview of the recent applications of infrared (IR) laser-induced temperature-jump (T-jump) and fast heat-conductive temperature-drop experiments on low-density lipoprotein dispersions can be found in [59,60]. Indeed, there are a number of emerging experimental setups which allows for the probing of not only the structural changes during phase transition, but also of other physical parameters. For instance, differential scanning calorimetry X-rays cells (DSC) have been developed to record scattering patterns simultaneously with enthalpy changes [61]. The development of SAXS coupled with other techniques for *in-situ* and real time characterization in non-equilibrium conditions is currently the route pursued to better exploit the properties of stimuli-responsive materials that can be used for solubilizing and controlling the release of drugs, peptides, and other bioactive materials on demand.

## 3. SANS for the Characterization of Liposome Nanocarriers

### 3.1. Pure Liposome

The large variety of molecules such as phospholipids, polypeptides, or more recently biocompatible block copolymers, used to formulate liposomes opens an immense domain for the formulation of liposome nanocarriers. Specific requisites such as the overall size, the monodispersity, the stability with time, temperature or under specific pH and ionic strength, are necessary before characterizing the drug loaded-liposomes. We do not pretend here to present an exhaustive list of publications but rather to give to the reader an idea of the contribution of the SANS technique to the field through few selected examples.

Uniform size self-assembled unilamellar vesicles (ULVs) can be produced from mixtures of weakly charged short- and long-chain phospholipids. In [62,63] the effect of various parameters—lipid concentration and composition, charge density and membrane rigidity on ULVs—was systematically investigated by SANS. Below the Krafft temperature, nanodisk-like aggregates are favored where the shorter chains form the rim of the disk. By increasing the temperature, the bicelles coalesce and then fold onto themselves, forming monodisperse vesicles with a total radius varying between 10 and 20 nm and a bilayer thicknesses from 2.3 to 3.9 nm.

Cholesterol is a main component of mammalian cells and plays a critical role in permeability, rigidity, dynamics and interaction with the cytoskeleton. Excess cholesterol can induce severe coronary pathology with the narrowing of the inner blood vessel walls through the deposition of plaques containing cholesterol crystals, which is due to the solubility limit of cholesterol into lipids. A recent SANS study performed on lipid contrast matched conditions to isolate the scattering coming from the cholesterol [64] inserted in a POPC or POPS liposome, has revealed a molar fraction solubility limit of 61% and 73% of cholesterol in POPC and POPS, respectively. Further, no evidence of stable nanodomains of cholesterol in POPS membranes was found, as suggested in other reports. These results are in agreement with coarse grained molecular dynamics simulations.

Liposome stability is a major requirement for drug delivery. Polysaccharides such as chitosan have been used to reinforce the stability, but also to improve bioadhesion and absorption [65,66]. The presence of trimethyl chitosan (TMC) does not affect the local structure of the DMPS/POPC bilayers. The amount of TMC is a key parameter to induce and control the multilayer structure and to tune the surface charge of the particles. Finally, the formulation that maximizes vesicle multilamellarity corresponds to that displaying the highest drug loading efficiency.

The grafting of polyethylene glycol (PEG) on the surface of liposomes is one of the most commonly applied ways of increasing both *in vitro* and *in vivo* stability of liposomes. The formed liposomes are commonly referred to as stealth liposomes because the PEG corona delays opsonization and phacocytosis and increases the circulation time in the bloodstream. The first detailed model is described in [67] “hairy” bilayer and allows for the extraction, beside the global size and the layer thickness, of the radius of gyration of the attached polymer coil. An example of linear or hyperbranched stealth DOPC-based liposomes and SANS characterization can be read in [68].

### 3.2. Effect of External Stimuli on the Liposome Structure

Synthetic amphiphilic polymers have been widely developed in order to form self-assembled polymer vesicles (polymersomes) which mimic liposomes. Polymersomes are more stable, more robust, and less permeable than liposomes thanks to the high molecular weight of polymers. Their properties can be tuned by a biochemical design of the building amphiphilic blocks to achieve a protective coat against rapid elimination by the immune system, site-specific ligands for targeting, and stimuli-responsive properties for controlled release. In [69], the effect of temperature on the structure of three different polymersomes has been investigated. The polymersomes are made of block copolymers containing a 2000 Da polyethylene glycol (PEG) as a hydrophilic block, and either a liquid-like polymer (e.g., PBA: polybutylacrylate), a solid-like polymer (PS: polystyrene), or a liquid crystalline (LC) polymer as a hydrophobic block. Above the critical dehydration temperature, the PEG cannot stabilize the initial bilayer vesicle structure and the PEG dehydration induces structural and morphological modification in polymersomes. Glassy PEG-*b*-PS polymersomes aggregate together above 55 °C, but the bilayer membrane is robust enough to remain intact. This aggregation is reversible, and rather separate polymersomes are recovered upon cooling. LC polymersomes display drastic and irreversible structural changes when heated above ~55 °C. These changes are dependent on the LC structures of the hydrophobic layer. Nematic LC polymersomes turn into thick-walled capsules, whereas smectic LC polymersomes collapse into dense aggregates. As these drastic and irreversible changes decrease or remove the inner compartment volume of the vesicle, LC polymersomes can be used for thermal-responsive controlled release.

Nanocarriers can be submitted to an osmotic imbalance when injected in physiological media leading to morphological changes. In [70], polymersomes were osmotically stressed and characterized by combined TEM, DLS and SANS. Hypotonic shock leads to a swelling of the vesicles, and hypertonic shock leads to collapsed structures such as unilamellar vesicles transform into bilamellar vesicles, a transition that might strongly affect the drug transport and delivery.

### 3.3. Drug-Loaded Liposome

A step further in the elaboration of nanocarriers consists of the insertion of active molecules. The final shape and size of liposome is a fine balance between attractive and repulsive molecular interactions and competition between the membrane bending properties and surface tension. The addition of host molecules may induce drastic changes in shape and size. Therefore, it is of primary importance to first verify that the liposomes are still present and, second, to locate the drug molecule. In [71], a novel ruthenium complex, linked to a cholesterol-containing nucleolipid (named ToThyCholRu), stabilized by POPC lipid aggregates for antineoplastic therapy, has been characterized. The resulting nanoaggregates contain up to 15% in moles of the ruthenium complex, and are shown to be stable for several weeks. The data obtained through SANS experiments, made it possible to demonstrate that the observed aggregates are liposomes. The thickness of the layer changes from ~4 nm for pure POPC liposomes to ~4.5 nm for ToThyCholRu-POPC (5:95), and to ~5 nm for ToThyCholRu-POPC (15:85), indicating that the housing of the ToThyCholRu in the bilayer induces a slight increase of its thickness. The liposomes host the ruthenium-nucleolipid complex with the metal ion surrounded by POPC lipid headgroups, and the steroid moiety inserted in the more external acyl chain region. It has been shown that these ruthenium-containing liposomes are more effective in inhibiting the growth of cancer cells.

A detailed combined SAXS-SANS characterization of loaded liposomes can be read in [11]. It concerns the incorporation of carboranes and their derivatives in liposomes made by dioleoylphosphatidylethanolamine and dimethyldioctadecylammonium bromide (DOTAP/DOPE). Carboranes are efficient boron delivery agents in boron neutron capture therapy, an anticancer treatment based on neutron absorption by ^10^B nuclei. Cationic liposomes were prepared using the positively charged DOTAP and the zwitterionic DOPE, these liposomes being used in gene therapy for their ability in targeting the cell nucleus. From the analysis of the scattering length densities profile of the bilayer, obtained from a simultaneous fitting of the SAXS and SANS curves in absolute scale, it is found that of the three carboranes investigated, only OCB, which does not possess a polar part, showed incomplete loading. Glucosyl carborane and lactosyl carborane are fully taken up by DOTAP/DOPE liposomes at a high molecular fraction (one carborane molecule for each lipid), and the sugar moieties are included in the polar head layer of liposomes. In the case of lactosyl, definite protrusion of the lactose double-ring is observed. In no case the original liposomes are disrupted and on the contrary, a substantial retention of their global size and shape is demonstrated. The results obtained in this work indicate that DOTAP/DOPE liposomes, loaded with sugar-derived carboranes, can be considered potential boron carriers for BNCT.

### 3.4. Time-Resolved SANS (Tr-SANS)

At the beginning of the 2000s, high-flux neutron sources have opened the possibility of carrying real-time measurements with very short acquisition times, on the order of a few tens of milliseconds. The most powerful SANS spectrometers—for example, D22 at the Institut Laue Langevin—have a maximum of flux of 10^8^ neutrons/s/cm^2^, whereas the maximum photon flux at the sample position at synchrotron sources can reach 10^14^ photons/s/mm^2^. The combined stopped-flow and neutron experiments started in the early 2000s, following noteworthy experiments that demonstrated the feasibility and potential of time-resolved SANS experiments [72].

The stopped-flow equipment, as with Tr-SAXS, is used to ensure a good reproducibility of the measurements and the synchronization between the mixture and the beginning of the acquisition, which is often necessary to repeat between five and 10 times to improve the statistics. The sample cell has been specially designed to match the geometry of the neutron beam. It consists of a relatively large quartz cell of 250 μL, which consequently induces a dead time during its filling and limits the observations of very fast processes shorter than 100 ms (Figure 2b).

Because of a larger availability of surfactant molecules, surfactant systems that mimics biological membranes are often used to investigate and understand the formation mechanism of vesicles. Two studies deserve to be quoted here. In [73], after mixing, the initial solutions of bilayer vesicles and micelles for DHDA and C12E12 respectively evolve into a mixed Lα (Lβ)/L1 phase. At 32 °C below the Lα/Lβ transition, the characteristic time is 130 min, and only on the order of seconds above the Lα/Lβ temperature; this indicates a drastic change in the monomer-exchange rate between the solid-like Lβ phase and the fluid-like Lα phase. In the second example [74], the formation of unilamellar vesicles by mixing zwitterionic tetradecyldimethylamine oxide (TDMAO) with the anionic lithium perfluorooctanesulfonate (LiPFOS) is studied as a function of the mixing ratio. Vesicle formation occurs via disk-like micelles as intermediates that close in order to form very monodisperse unilamellar vesicles after reaching a certain size. This process is controlled by the competition between bending energy of the bilayer and the line energy of the disk rim, and its characteristic time is controlled by the electrostatic repulsion that is determined by the TDMAO/LiPFOS ratio.

DNA and cationic liposomes associate to form complexes that are promising for the transfer of genetic material to cells. The association of DNA to DOPE/DODAB (dioleoylphosphatidylethanolamine and dimethyldioctadecylammonium bromide) unilamellar vesicles investigated by Tr-SANS with a time resolution from one to several seconds [75] reveals a multistep process. In a few milliseconds, the electrostatic interactions drive the coverage of the DNA strand by one single vesicle, which induces the breakdown of the latter. In a time scale of seconds, the bilayers roll up around the DNA long axis. A layer-by-layer association of the intermediate cylinders yields to a multilamellar structure after a few minutes.

In a recent study [76], the insertion mechanism of the antifungal drug amphotericin B and formulate derivative with sodium cholesteryl sulfate (SCS) in ergosterol-containing (model fungal cell) and cholesterol-containing (model mammalian cell) membranes has been investigated. stopped-flow SANS experiments reveal that the structural changes to vesicle membranes occur far more rapidly following exposure to AmB-SCS *vs*. free drug with the kinetics of the changes varying with membrane composition. A membrane swelling of 0.4 nm is observed in both cases, but the equilibrium state is reached within 10 s for ergosterol and 120 s for cholesterol, in agreement with the known differences in affinity of AmB for these two sterols.

## 4. Conclusions and Perspectives

The present overview on small-angle scattering with synchrotron and neutron sources illustrates how SAXS and SANS can be used as valuable tools to obtain quantitative information about the internal organization and the functioning mechanisms of engineered pharmaceutical formulations with high resolution. The difference in contrast mechanisms makes these two techniques highly complementary for getting the full description of multi-component systems. Moreover, the parallel analysis of SAXS and SANS spectra, if performed at the absolute scale, allows quantitative modelling of the detailed structures of plain and loaded nanovectors. SANS contrast variation can help to enhance or decrease the scattering contribution from specific regions (polar heads, the hydrophobic compartments or other functional groups) in lipid-based aggregates. The method has been successfully used to shed light on fundamental aspects concerning liposomes, such as lipid sorting, in the bilayer and the loading modality of biomolecules or drugs. More recently, ASAXS has also shown to be a suitable method to localize guest molecules in liposome systems, though direct modelling of the scattering curves requires that the anomalous effects be rather pronounced.

The high flux provided by modern instruments makes it possible to obtain a complete scattering profile in a short time (down to few milliseconds). Therefore, Tr-SAXS/SANS techniques can also be used to analyze relatively unstable systems, such as transient conformations in evolving structures or relatively unstable formulations, whose evolution pathway can be of interest for various purposes.

It is therefore desirable that the knowledge and practice of SAXS and SANS is spread to a larger research community in the biomedical and pharmaceutical fields, especially for those studies where advanced tools are requested to refine the information on function-structure relationships that can be obtained by routine methods.
